# Step Counting: A Review of Measurement Considerations and Health-Related Applications

**DOI:** 10.1007/s40279-016-0663-1

**Published:** 2016-12-22

**Authors:** David R. Bassett, Lindsay P. Toth, Samuel R. LaMunion, Scott E. Crouter

**Affiliations:** Department of Kinesiology, Recreation and Sport Studies, University of Tennessee, Knoxville, 1914 Andy Holt Ave., Knoxville, TN 37996 USA

## Abstract

Step counting has long been used as a method of measuring distance. Starting in the mid-1900s, researchers became interested in using steps per day to quantify ambulatory physical activity. This line of research gained momentum after 1995, with the introduction of reasonably accurate spring-levered pedometers with digital displays. Since 2010, the use of accelerometer-based “activity trackers” by private citizens has skyrocketed. Steps have several advantages as a metric for assessing physical activity: they are intuitive, easy to measure, objective, and they represent a fundamental unit of human ambulatory activity. However, since they measure a human behavior, they have inherent biological variability; this means that measurements must be made over 3–7 days to attain valid and reliable estimates. There are many different kinds of step counters, designed to be worn on various sites on the body; all of these devices have strengths and limitations. In cross-sectional studies, strong associations between steps per day and health variables have been documented. Currently, at least eight prospective, longitudinal studies using accelerometers are being conducted that may help to establish dose–response relationships between steps/day and health outcomes. Longitudinal interventions using step counters have shown that they can help inactive individuals to increase by 2500 steps per day. Step counting is useful for surveillance, and studies have been conducted in a number of countries around the world. Future challenges include the need to establish testing protocols and accuracy standards, and to decide upon the best placement sites. These challenges should be addressed in order to achieve harmonization between studies, and to accurately quantify dose–response relationships.

## Key Points

**Table:** 

Steps are a fundamental unit of human locomotion, and thus are a preferred metric for quantifying physical activity.
In cross-sectional studies, strong associations between steps per day and health variables have been documented.
Many step-counting devices are available for both consumer and research use, but the need for industry standardization is acknowledged and must be addressed in order to harmonize data.

## Introduction and Usage

Step counters are devices worn on the body that measure steps and/or distance traveled. The original purpose of these devices was to measure distance traveled, when walking was the most common mode of transportation. As early as 1960, researchers have been interested in using step counters to assess physical activity [1]. In the 1990s, the use of step counters to measure physical activity and study relationships between physical activity and health began in earnest [2]. Since 2011, interest in step counting has exploded within the general population, as people have become fascinated with tracking their levels of physical activity. This is part of a larger movement known as “the quantified self” [3] in which people are seeking to gain knowledge through numbers, and using technology to acquire data on aspects of a person’s daily life in terms of physiological variables, environmental exposures, and psychological mood states.

In recent years, the popularity of activity trackers that count steps has grown substantially. A single company, Fitbit, has experienced exponential growth, and sold 21.4 million devices worldwide in 2015 [4] (Table 1). Using the search terms “pedometer” and “activity tracker,” Amazon and Walmart listed 181 and 139 different devices, respectively, on their websites (13 July 2016). These activity trackers may provide estimates of steps, calories, distance traveled, time in activity, and “wear time.” While consumer interest has increased in recent years, there is a problem in that the accuracy of these devices is not regulated by any government agency or scientific body to ensure that they are giving valid information. To fill this void, the Consumer Technology Association (CTA) formed a Health and Fitness Technology Division in 2010. In 2016, they hosted a Medical Advisory Summit to bring together key players in the technology and medical fields, for the purpose of having a forum to develop standards for wearable devices to track physical activity. The CTA is attempting to develop best-practice testing protocols and voluntary standards that companies can meet in order to achieve data quality (i.e., performance benchmarks).

**Table 1:** Number of Fitbit devices sold worldwide from 2010 to 2015. From Statista [4]

Year	No. of devices sold per year (in thousands)
2010	58
2011	208
2012	1279
2013	4476
2014	10,904
2015	21,355

## History of Step Counting

Step counting began as a method of estimating distance. Thus, it is a logical extension of other measurement methods based on the human body, including the inch (i.e., width of thumb), the hand (i.e., width of the palm), the foot (i.e., length of the foot), the cubit (i.e., distance from elbow to fingertip), and the fathom (i.e., distance between fingertips with arms outstretched). The word mile comes from the Latin phrase *milia passuum*, meaning “one thousand paces.” The Roman mile was approximately 1000 paces (or 2000 steps) of a full-grown adult [5].

Leonardo da Vinci is credited with inventing the first mechanical step counter. It was worn at the waist, with a long lever arm that was tied to the thigh. When the thigh moved back and forth in walking, the gears were rotated, causing steps to be counted [6].

Thomas Jefferson commissioned a step counter made by one of the best watch-makers in Paris. It was worn in a vest pocket, and had a lever arm which was tied to a string that passed through a hole in the bottom of the vest pocket. The other end of the string was tied to a strap below the knee, and walking caused it to pull on a lever arm attached to gears. He used his pedometer to measure out the distance to Paris landmarks in steps. Jefferson noted an English mile would require 2066.5 steps, while the brisk walk of winter reduced it to 1735 steps [7]. He sent a pedometer to James Madison in 1788 along with a detailed one-page letter of instructions [8].

In 1777, Abraham-Louis Perrelet, a Swiss-born watchmaker invented a self-winding mechanism for pocket watches that used an oscillating weight inside the watch that moved up-and-down during walking. In 1780 he invented a self-contained pedometer that also used a spring-suspended lever arm to count steps [9]. In 1820, Abraham-Louis Breget designed a mechanical pedometer/stopwatch for Alexandre I, Tsar of Russia, for use in measuring the distance and pace of his marching armies [10].

The Yamasa company in Tokyo, Japan (internationally known as Yamax) designed a *manpo*-*kei* (10,000 steps meter) in 1965 [11]. The 10,000 steps per day slogan originated in Japan around 1965, shortly after the Tokyo Olympics. This was believed to be the amount of physical activity that would be sufficient to decrease the risk of coronary heart disease. The Yamasa company continually refined their step counter, adding a mechanism to prevent double-counting of steps in 1987 [11]. Around 1990, Yamasa introduced the Digi-walker (DW-500) containing a hair-spring suspended lever arm, an electronic event counter, and a digital display [12].

Since 1996, quantifying steps has become an accepted method of assessing physical activity in scientific research. One pedometer (Yamax DW-500) was found to be more accurate and reliable than others [12]. A few years later, this step counter was used to validate questions about walking distance on physical activity questionnaires [13]. At about this time, other researchers began using pedometers for population surveillance [14] and walking interventions [15].

## Types of Step Counters

There are many different types of step counters. They fall into five general categories, based on where they are worn on the body, and the internal mechanism (spring-suspended lever arm vs. accelerometer) used to record steps. In this section, we review the mechanisms, accuracy, and sources of error for the various types of step counters.

### Waist-Worn, Spring-Levered

The traditional step counter was designed to be worn at the waist, attached to the belt or waistband. The most basic type uses a mechanical internal mechanism. In walking or running, the vertical accelerations of the body cause the horizontal, spring-suspended lever-arm to move up and down with each step. The movement of the lever arm opens and closes an electrical circuit, causing an electronic counting device to register steps. In the case of the Yamax pedometer, every movement of the trunk that exceeds the vertical acceleration threshold of 0.35 g is considered a step [16]. Whenever the threshold is exceeded, it results in an event being recorded.

The main sources of error for this class of devices are slow walking speeds and obesity, which both result in underestimation of steps. Studies have demonstrated that most waist-mounted pedometers are very accurate at speeds of 3.0 mph (80.4 m/min) and above, but their accuracy declines at slower speeds. At 2.0 mph (54 m/min) they may capture 75% of steps, and at 1.0 mph they hardly register steps at all. Thus, waist-mounted pedometers are notoriously inaccurate in older adults in assisted-care settings, who walk with a slow, shuffling gait [17]. Double-counting of steps is a common problem in inexpensive pedometers, if care is not taken to prevent it. Spring-levered pedometers have diminished accuracy in obese individuals. According to Crouter et al. [18], this is because when they are tilted away from the vertical axis their sensitivity is diminished, causing them to undercount steps.

### Waist-Worn, Accelerometer

More recent waist-mounted step counters use an internal mechanism consisting of a piezoelectric or piezo-resistive accelerometer (typically tri-axial). In walking or running, there is a sinusoidal pattern of acceleration with both positive and negative accelerations being recorded during various phases of the ambulatory cycle. With this type of step counter, the number of zero crossings or peaks of the vertical acceleration of the body versus time curve is used to determine the number of steps. The Omron HJ-720, the New Lifestyles NL-2000, the Fitbit One, and the Fitbit Zip are examples of this type of pedometer.

Waist-worn accelerometer-based step counters are generally more accurate than spring-levered pedometers. Two such devices (New Lifestyles NL-2000 and Omron HJ-720) are not impacted by obesity or tilt angle [18, 19]. However, these devices still show a tendency for diminished accuracy at slow walking speeds.

### Pocket

Some activity trackers can be worn in the pants pocket, including the Omron HJ-720, Phillips DirectLife, Fitbit Zip, and Misfit Shine. Similar to the waist-worn devices, these monitors have triaxial accelerometers that detect accelerations of the body during walking and running.

Major sources of error are basically similar to those of waist-worn, accelerometer-based devices. Also, in the case of the Omron, steps taken in brief walking bouts go undetected because of the presence of a 4-s filter [20].

### Thigh

The activPAL monitor is designed to be taped to the thigh. This device uses a uni-axial accelerometer which responds to gravitational acceleration as well as the accelerations resulting from leg movements. The accelerations that occur during walking and running are used to count steps. Data are stored in memory, time-stamped, and can later be downloaded to a computer for subsequent recall.

The activPAL is useful as a tracking device only, since it has no data display for providing biofeedback to participants. It accurately counts steps down to 1.5 mph (40.2 m/min) [21] and only underestimates steps by 3.5% at 1.0 mph (26.8 m/min) [22].

### Ankle

The most accurate step counter for walking is the StepWatch 3 device, worn on the ankle [23, 24]. It contains an analog accelerometer (not a micro-electrical mechanical system or MEMS accelerometer) that samples a 120 Hz data stream of acceleration. The StepWatch is able to detect several signature movements involved in stepping, ensuring that it has high sensitivity and specificity for recording steps.

The StepWatch is accurate to within 1–2% of actual steps, even at very slow walking speeds, and even in individuals who are obese [25]. In addition, Hickey et al. [26] have shown that this device is even accurate for housework activities like dusting, filing, and cleaning. However, the StepWatch will record extra steps if the user performs bicycling, heel tapping, or leg swinging [24]. In addition, the StepWatch undercounts steps in running, when programmed with the default settings [26].

### Foot

Shoe-mounted step counters are designed so that contact of the heel with the ground causes a step to be recorded. Some fit on the shoe laces. Another type has a pressure transducer, circuitry and rechargeable battery are placed into the heel of a normal shoe and can detect when the heel is in contact with the ground [27]. This shoe-mounted device is consistent with defining a step as any time the foot is lifted up off the ground and put back down again. This latter type was tested in patients with chronic health failure and healthy age-matched volunteers, and found to be more accurate than body-worn step counters. The sources of error with foot step counters have not been investigated, but they most likely exhibit the same errors as ankle step counters.

### Wrist

Recently, wrist-worn activity trackers have been designed that measure steps (e.g., Nike Fuelband, Jawbone UP, Garmin VivoFit, Fitbit Flex, Fitbit Surge, Fitbit Charge, Misfit Shine, Polar A360, Polar Loop, etc.). At first glance, it may seem illogical to place a device on the wrist in order to assess steps taken by the feet. However, a study by Chen et al. [28] reported that three wrist devices (Fitbit flex, Garmin Vivofit, and Jawbone Up) were quite accurate (absolute percent error for steps = 1.5–9.6%) during treadmill walking and running at 54–134 m/min. Smartwatches such as the Apple Watch, Samsung Gear S2, and Pebble Watch are also reported to have acceptable validity and reliability, at least for measuring steps during overground walking [29]. Their accuracy for counting steps during activities of daily life has not been studied.

Wrist step counters do not count steps when the wrist is stationary. For example, they do not record steps taken when pushing a stroller [28], or holding onto treadmill hand rails. Furthermore, wrist-worn step counters record invalid steps when folding laundry [28], or gesturing while talking. These sources of error are troubling to physical activity researchers who are focused on obtaining accurate step counts.

The US National Health and Nutrition Examination Survey, or NHANES (2008–2014) used an Actigraph GT3X+ worn on the non-dominant wrist. The previous deployment of the Actigraph 7164 in NHANES (2003–2006) had used the waist location. The wrist placement site and a waterproof case increased wear times, and had the added benefit of providing a valid assessment of sleep duration and quality [30]. Unfortunately, the step detection algorithm developed for the waist does not seem to work for the wrist location. Tudor-Locke et al. [31] examined the accuracy of the wrist and waist attachment sites for the ActiGraph GT3X+. Compared to directly observed steps, the waist site performed better than the wrist site at most treadmill speeds, regardless of the bandpass filter. However, in the free-living environment the wrist recorded more steps than the waist, likely due to extraneous arm movements. In the free-living environment, the waist-worn ActiGraph measured 6743 ± 2398 (default filter) and 13,029 ± 3734 (low-frequency extension) steps per day. The wrist ActiGraph measured 9301 ± 2887 (default filter) and 15,493 ± 3958 (low-frequency extension) steps per day. ActiGraph is working to improve their step counting algorithm for the wrist (John Schneider, ActiGraph L.L.C., personal communication, 6/23/2016).

In summary, there are various ways of defining and measuring a step. When researchers seek to determine the accuracy of a device for step counting, it is important to select a criterion measure that is consistent with both of these. For many purposes, visual observation and hand-tally of steps by a trained investigator can serve as a valid criterion.

## Why Count Steps?

Tryon [32] has noted that steps are a fundamental unit of human locomotion, and thus are a preferred metric for quantifying physical activity. Measurement of steps has a number of other advantages:

- Steps are intuitive, and readily understandable to the layperson
- Steps can be measured easily and accurately
- Steps are objective
- Steps can be used to place people into less active and more active categories
- Steps/day has strong associations with physical health variables
- Steps are motivational, and they facilitate behavior change
- Steps have the potential to be useful in translating scientific results into public health messages.

## Classification of Steps per Day

Pedometers can be used as an overall index of how active a person is. Tudor-Locke and Bassett [33] proposed a classification scheme for categorizing adults based on their daily steps (Table 2). These categories were developed by taking descriptive data on steps per day, and thinking of terms that characterized groups based on perceptions of their activity levels. With recent studies showing that individuals who take more steps per day have more favorable cardiometabolic risk profiles, in the future it may be possible to develop criterion-referenced standards for steps per day and assign terms that refer to disease risk.

**Table 2:** Steps-per-day categories and classification system of Tudor-Locke and Bassett [33]

Steps per day	Classification
<5000	Sedentary lifestyle
5000–7499	Physically inactive
7500–9999	Moderately active
≥10,000	Physically active
≥12,500	Very active

Steps differ from other units of measurement. The scientific community has adopted *le Système International d’Unités* (SI units) in an attempt to reduce confusion in scientific writing. SI units provide a consistent system to express scientific data on physical quantities (centimeters, grams, seconds, etc.), to facilitate the exchange of information. However, steps are a behavior rather than an object or event. Thus, the step is an “anthropometric” unit of measurement, they cannot be quantified by absolute units like meters or kilojoule.

**Fig. 1:**
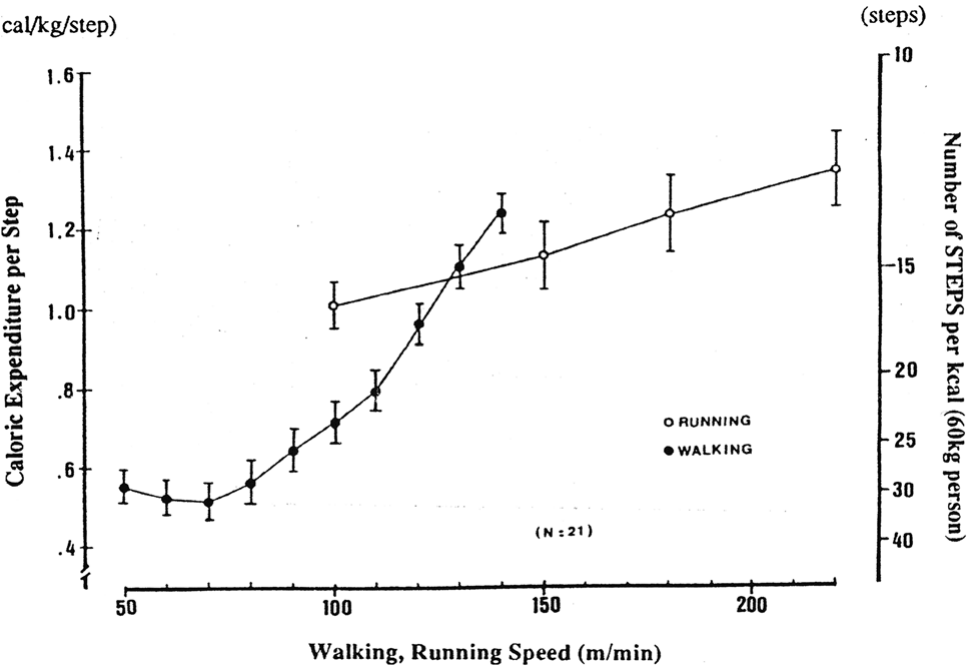
Relationship between locomotive speeds and rates of caloric expenditure. Reproduced from Hatano et al. [34] with permission

The steps that a person takes vary according to his/her height, age, and fitness level. The length of a walking step, at a self-selected pace, is roughly proportional to a person’s height (i.e., approximately 42% of height) [34]. The amount of energy expended per step is roughly proportional to a person’s body weight (cal/kg/step) [34], although it is also dependent upon speed of locomotion and whether one is walking or running (Fig. 1). Finally, the intensity of steps can vary with one’s level of aerobic fitness. Frail, elderly individuals tend to take slower, shorter steps while younger, more athletic individuals often take running steps. This is consistent with differences in physical work capacity and aerobic fitness across the age span.

## What is a Step?

Merriam-Webster defines a step as “a movement made by lifting your foot and putting it down in a different place” [35]. (Marching in place could also be considered stepping, though it does not fit this definition.) Researchers working in the field of human gait and rehabilitation sometimes broaden this definition of a step to include a prosthetic device that takes the place of a foot. So, a step can be defined as any time the foot or prosthetic device is lifted off the ground and put back down again, in the process of ambulating.

The Oxford dictionary defines a step as “an act or movement of putting one leg in front of the other in walking or running” [36]. Note that this definition implies that a step needs to be part of a sequence of similar events that make up a continuous walking or running bout. Some researchers believe that a minimum walking bout requires that several steps be taken [37].

Some researchers define a step as an event that occurs when the foot or prosthetic device is unweighted, moved to a new location, and then re-weighted, in the load path of the body (Michael Orendurff, personal communication, 4 March 2012). This definition acknowledges that frail, elderly individuals often take “shuffling” steps in which the foot is not lifted all the way off the ground, but rather moved forward while maintaining contact with the ground. Even in healthy people, certain activities (e.g., waltz, tango, and tennis) may involve “sliding” the foot from one location to another. According to the first two definitions discussed, shuffling and sliding events would not be classified as steps.

Some ankle-mounted accelerometers detect forward accelerations of the foot during the swing phase. This measurement method is consistent with defining a step as any time the foot is moved ahead of the opposite foot into a position to accept weight transfer from the other limb (i.e., the “load path”) and then put down again. Interestingly, people with Parkinson’s disease may advance the lower limb while it is still bearing 1–5% of body weight, but this is considered a step as long as the opposite limb bears greater than 50% of body weight. By this definition, one foot would be in stance phase and the other would be in swing phase, even if the swing limb is dragging across the floor.

It is interesting to consider all the different kinds of steps humans take. There are forward steps, backward steps, side-to-side steps, diagonal steps, puttering steps, walking steps, and running steps. Subtle movements to reposition the body, for example when cooking meals in the kitchen, can result in steps even though there is not necessarily forward movement of the body. With the advent of data-storing pedometers, it is harder to defend pedometers that undercount steps during slow walking and intermittent, lifestyle activities. We advocate for counting all steps, and then distinguishing between various types of steps (e.g., those taken at light, moderate, or vigorous intensity or those taken in continuous vs. intermittent walking bouts) through various analytical procedures.

Biomechanical studies show that in walking and running, each leg has a stance phase corresponding to the period of time when the foot is on the ground, and a swing phase corresponding to the period of time when the foot is off the ground and moving forward. In walking, there is a double-stance period where both legs are on the ground, but in running this does not occur. In the sport of race walking, judges use this to determine if participants are walking or running, and the failure to exhibit double-stance will result in disqualification of a race walker.

## Manufacturer’s Solutions for Reducing Pedometer Error

The preceding section identified sources of error for various types of pedometers. In general, these sources of error will either result in overcounting or undercounting of steps. In an effort to prevent these errors, pedometer manufacturers have attempted to devise solutions to enhance pedometer accuracy.

Undercounting at slow speeds is a problem that afflicts most step counters. The Japanese Ministry of Economy Trade and Industry has set industrial standards for pedometers (they must be accurate to within ±3% for approval) [11]. Good step counters can record steps within this range during walking at 80.4 m/min, or 3 mph [12, 18, 24, 38, 39]. However, at 54 m/min (2 mph) many devices begin to undercount steps, and at 26.8 m/min (1 mph) most devices will record only 50–75% of actual steps. Only a few research grade step counters (i.e., StepWatch, activPAL) can accurately record steps at 1.0 mph, and these devices cost around US$500 each. These devices are ideal for use in frail, older individuals and in clinical populations with disabilities. The StepWatch device actually permits researchers to customize the cadence and sensitivity settings for individual users. A researcher can select various options that describe the user’s walking speed, leg motions, quick stepping, and range of speeds.

Double-counting of steps is a common problem with less expensive, spring-levered step counters. As mentioned previously, the Yamasa corporation discovered a way to prevent this in 1987. Their DW-500 step counter will not record an event as a step, if it follows too closely in the wake of a preceding step. This “refractory period” is one solution to preventing double counting. The Yamax SW-200 model has a pivoting head on the spring-suspended pendulum, and conductive rubber sheaths on the electrical contact posts, which absorb energy. This is a mechanical solution to prevent double-counting of steps.

When a step counter is jostled or exposed to mechanical vibrations, erroneous steps can be recorded even though none are taken. This problem is especially common with wrist devices, because wrist movements occur during housework or talking with gesturing. This problem is evident with Fitbit wrist-worn devices, as users report accumulating steps during teeth brushing, eating, gesturing, etc. A partial solution is to put the device on the non-dominant wrist, since the dominant hand is used for many activities that may result in erroneous steps. A second partial solution is to decrease the sensitivity (i.e., raise the threshold acceleration need to detect a step) when the device is worn on the dominant wrist.

To further reduce erroneous steps, some devices (e.g., Omron step counters) use a 4-s filter and will not count any steps unless the user walks/runs for at least 4 s. A regular, rhythmic pattern of stepping must be detected for step counting to occur. This has the advantage of eliminating some “false-positive” steps, but unfortunately it also eliminates some actual steps in short walking bouts. Orendurff [37] showed that the most common walking bouts last only four steps, and the next most common bouts last six, eight, ten, 12 steps, etc. This is one reason why the Omron HJ720 gives lower step counts than other pedometers, because it has a 4-s filter that results in failure to record steps during intermittent, lifestyle activities.

## Steps/Day and Health

### Cross-Sectional Studies

Cross-sectional studies have shown that daily step counts are inversely related to body mass index (BMI), hypertension, and diabetes [11, 40]. Thus, there has been interest in the variable “steps per day” as an overall measure of physical activity. However, this metric does not tell us the frequency, intensity, or duration of a person’s physical activity bouts.

Schmidt et al. [41] analyzed data from the Childhood Determinants of Adult Health study (*N* = 1793) and the Tasmanian Older Adult Cohort Study (*N* = 1014); both studies were done in Australia. They measured the prevalence of metabolic syndrome in younger and older adults who wore a pedometer for 7 days. Except for younger men, individuals who took ≥5000 steps per day had a lower prevalence of metabolic syndrome than those who obtained fewer steps. Among the higher step categories, the differences in cardiometabolic risk factors were modest. However, younger men and women in the highest step category (12,500 steps per day) had a substantially lower prevalence of cardiometabolic risk those who took fewer steps per day (Table 3).

**Table 3:** Steps-per-day categories and prevalence of metabolic syndrome in Australian men and women. From Schmidt et al. [41]

Activity level	Sample		Metabolic syndrome		
	*N*	%	% with MetS	PR	95% CI
Men					
Sedentary (0–4999)	69	7.8	13.0	1.00	Ref
Low-active (5000–7499)	247	27.9	14.6	1.22	0.62–2.39
Somewhat active (7500–9999)	242	27.3	12.4	0.98	0.49–1.95
Active (10,000–12,500)	190	21.4	10.5	0.72	0.34–1.51
High-active (≥12,500)	139	15.7	4.3	0.29	0.11–0.79
*P*_trend_			<0.01	<0.001	
Women					
Sedentary (0–4999)	56	6.2	14.3	1.00	Ref
Low-active (5000–7499)	253	27.9	5.5	0.39	0.17–0.86
Somewhat active (7500–9999)	301	33.2	4.0	0.30	0.13–0.70
Active (10,000–12,500)	193	21.3	6.2	0.48	0.21–1.10
High-active (≥12,500)	103	11.4	2.9	0.22	0.06–0.79
*P*_trend_			0.06	0.10	

Sisson et al. [42] analyzed data from 1446 adults in the NHANES study (2005–2006). This study used the Actigraph 7164 worn at the waist, and the authors “censored” steps taken at lower intensity levels. This procedure involved not counting the steps taken during any minute in which the accelerometer recorded fewer than 500 activity counts/min. This was done in order to make the Actigraph step counts more similar to those obtained with a Yamax Digi-walker. For each 1000-step increase in steps per day, the prevalence of metabolic syndrome was 10% lower. The likelihood of metabolic syndrome was OR = 0.28 (95% CI 0.18–0.44) for those in the active to highly active categories, compared to those who were sedentary. The likelihood of metabolic syndrome was OR = 0.60 (95% CI 0.43–0.82) for those in the low to somewhat active categories.

Inoue et al. [43] examined 1166 men and 1453 women (40–64 years of age) in the National Health and Nutrition Survey of Japan, 2006. They used a Yamasa (Yamax) AS-200 pedometer which participants were instructed to wear during all waking hours for a single day, except when bathing and showering. In men, inverse associations were noted between steps per day and cardiometabolic risk factors. In women, those taking ≥5000 steps per day had substantially lower likelihood of overweight obesity and hypertension than women taking fewer steps; however, further increases in steps/day were only accompanied by modest decreases in odds ratios. The authors noted that given the limitations of cross-sectional studies, longitudinal studies are needed to more precisely calibrate the impact of daily steps on CVD risk.

Katzmarzyk et al. [44] are conducting the International Study of Childhood Obesity, Lifestyle, and the Environment (ISCOLE). The major purpose is to determine the relationships between lifestyle behaviors and obesity in a multinational study of children. The study sample includes children 9–11 years of age (*N* = 6000) from 12 nations in diverse geographic regions (Europe, Africa, the Americas, South-East Asia, and the Western Pacific). An ActiGraph GT3X+ accelerometer was worn at the waist, and children were encouraged to wear it 24 h per day for 7 days. Since the ActiGraph provides a measure of steps, it is likely that this group will report on associations between steps per day and obesity in their study sample.

Despite the potential utility of steps per day for translating research findings into public health recommendations, step guidelines (e.g., 10,000 steps per day) have not been widely adopted by government health agencies. However, the US President’s Council on Fitness, Sports, and Nutrition has set daily step goals as part of its President’s Active Lifestyle Award (PALA+): 12,000 steps per day for youth aged 6–17 years, and 8500 steps per day for adults [45].

### Prospective Observational Studies

Prospective, observational studies are currently being conducted that use wearable monitors to assess physical activity in large cohorts (Table 4). These studies will take 5–10 years before the results are in, but they will provide evidence on the dose–response relationships with physical activity and all-cause, cardiovascular disease, and cancer mortality. Since the researchers are using accelerometers, they will have an opportunity to express physical activity with various metrics, including minutes of moderate-to-vigorous physical activity (MVPA), kilocalories, and steps. This highlights the importance of accurate step counting, and the need to harmonize step data coming from different wearable devices.

**Table 4:** Prospective, longitudinal studies using wearable activity monitors to assess physical activity and examine it in relation to disease endpoints. Reproduced with permission from Dr. I-Min Lee (*Wearable Devices and the 24*-*Hour Activity Cycle*, conference held at Stanford University, Palo Alto, 27–28 April 2016)

Study	Start year	Sample size	Population age	Device	Delivery mode
REGARDS	2008	~10,000	56+ years	Actical	Mail
EPIC Norfolk	2008	3892	60–80 years	ActiGraph GT1 M	In Person
Actife Ulm	2009	1500	65–90 years	ActivPAL	In Person
BRHS	2010	~2500	Mean 78 years	ActiGraph GT3X	Mail
Maastricht Study	2010	~10,000	40–75 years	ActivPAL	In Person
WHS	2011	~18,000	62+ years	ActiGraph GT3X+	Mail
WHI	2012	~7000	63+ years	ActiGraph GT3X+	In Person/Mail
UK Biobank	2013	~100,000	40–69 years	Axivity AX3	Mail

*REGARDS* reasons for geographic and racial differences in stroke, *EPIC* European prospective investigation of cancer, *BRHS* British Regional Heart Study, *WHS* Women’s Health Study, *WHI* women’s health initiative

### Longitudinal Intervention Studies

Beginning in the mid-1990s, step counters began to be used in behavioral interventions designed to increase physical activity in inactive, outpatient adults. Several excellent, comprehensive reviews have been conducted that summarize this research [15, 46, 47]. Bravata et al. [15] identified eight randomized controlled trials (RCTs) and 18 studies without a control group that used step counters in an attempt to increase physical activity. In the RCTs, pedometer users increased their physical activity by 2491 steps per day more than individuals assigned to control groups. Among the studies that lacked a control group, pedometer users significantly increased their physical activity by 2183 steps per day over their baseline values. Having a step goal and keeping a physical activity log were other elements found to be helpful in increasing physical activity. When data from all studies were combined, pedometer users reduced their BMI by 0.38 kg m^−2^ (95% CI 0.05–0.72 kg m^−2^, *P* = 0.03), and their systolic blood pressure by 3.8 mmHg (95% CI 1.7–5.9 mmHg, *P* < 0.001).

Richardson et al. [46] conducted a meta-analysis of step-counting interventions without a dietary intervention that reported weight change as an outcome. They sought out randomized controlled trials (RCTs) and prospective cohort studies published after 1995, and nine studies met all of their inclusion criteria. The duration of the interventions ranged from 4 to 52 weeks, with a mean duration of 16 weeks. The pooled estimate of the average weight change was −1.29 kg (95% CI −1.85 to −0.70 kg). On average, participants lost 0.05 kg per week. The authors concluded that pedometer-based programs result in modest weight loss, and that longer programs resulted in greater weight loss than shorter ones.

Kang et al. [47] conducted a meta-analysis of step counting interventions in 2009. They used six databases and searched for studies with the following inclusion criteria: (a) One or more groups used pedometers daily, (b) pedometers were used to motivate participants to increase their activity, (c) step counts were determined pre- and post-intervention, and (d) the intervention period lasted 4 weeks or more. They found 103 articles, and narrowed this down to 50 studies that met the inclusion criteria; however, some studies did not provide sufficient data to compute an effect size. For the remaining 32 studies, the overall mean effect size was 0.62, corresponding to an average increase of 2000 steps per day in the intervention group. Greater effects were seen in females, and with the use of a 10,000 steps per day goal. The authors concluded that step counters are associated with an increase in physical activity in intervention studies.

A recent viewpoint in the *Journal of the American Medical Association* concluded that wearable devices (e.g., step counters) are facilitators, not drivers, of behavior change [48]. The authors note that the use of wearable devices for effective physical activity promotion is a complex, multi-step process. First, the users must be motivated to want such a device and must be able to afford it. Second, once they have attained a device they must remember to wear it and occasionally recharge it. (Some devices must be synched with a smart phone or computer in order to download the data, and users must be motivated enough to do this.) Third, the device must be accurate in tracking the desired behavior. Finally, the data must be presented to the user in a format that is understandable, motivates action to change the behavior, and sustainably motivating. This may involve behavior change principles such as goal setting, overcoming barriers, extrinsic rewards, social support, and accountability. In fact, many consumer-based activity trackers are now incorporating such principles into their “apps,” although the effectiveness of these apps, compared to face-to-face delivery of physical activity interventions is unknown.

Recently, a number of studies have been conducted using pedometer programs, some delivered over the internet. For example, Richardson and co-workers have conducted successful pedometer studies in patients with type 2 diabetes [49], chronic obstructive pulmonary disease [50], low-back pain [51], and breast cancer [52] in Michigan. Other researchers are conducting similar interventions in clinical populations. Kaiser Permanente adopted the 10,000 steps pedometer program originally developed by Minnesota Health Partners [53], for use in a managed care setting [54].

In addition, a number of worksite wellness programs now use data-storing step counters to track employee’s physical activity (Virgin HealthMiles, Walkingspree, Walker Tracker, 10 K-a-Day, etc.) Some of these programs may lack an element of personal accountability to a researcher that has usually been present in most of the published research on pedometer interventions. However, by substituting phone calls and emails in place of personal face-to-face contact it appears that they are effective at increasing physical activity. In the future, more research is needed to determine the health outcomes and cost-effectiveness of such programs.

## Step Counting for Physical Activity Surveillance

The first use of pedometers for physical activity surveillance was conducted in Switzerland in 1988–1989. Sequira et al. [14] assessed a representative population sample of 493 men and women aged 25–74 years of age, taking part in the World Health Organization Monitoring Trends and Determinants in Cardiovascular Disease (MONICA) study. They used the Pedoboy, a small, low-cost, mechanical step counter made in Germany. The step counter was worn for 1 week, and the average number of steps per day decreased from 11,900 to 6700 and from 9300 to 7300 for men and women, respectively, in the youngest to the oldest age categories. Thus, males tended to take more steps than women, except in the oldest age categories. Occupation was also found to be associated with daily steps.

McCormack et al. [55] studied the physical activity levels of adults in Western in 2002. A subset of the original 3200 participants agreed to take part in the pedometer study. After completing a telephone interview, 603 out of 1326 individuals who were asked to wear a Yamax SW-700 pedometer for 7 days agreed to participate (45% response rate). On average, Western Australian adults took 9695 steps per day, and less than half (47%) took 10,000 or more steps per day. Men accumulated more steps per day (10,221 steps) than women (9178 steps), and younger adults accumulated more steps per day than older adults.

Inoue et al. [56, 57] analyzed the pedometer data resulting from the National Health and Nutrition Survey of Japan, conducted in 2003. This annual survey has been conducted by the Ministry of Health, Labor, and Welfare since 1945, and the Yamasa Digi-walker pedometer steps since 1992. In November 2003, 1-day step counts were administered in a nationally representative sample of 8867 individuals. On average (mean ± SD), Japanese residents 15 years of age and older took 7168 ± 4248 steps per day; males accumulated 7575 ± 4580 steps per day and women accumulated 6821 ± 3909 steps per day. Similar to other countries, there was an age-related decline in daily steps.

Tudor-Locke et al. [16] examined accelerometer-determined steps per day in United States Adults, using data from NHANES (2005–2006). They reported data on 3744 participants 20 years of age or older had at least one valid day with 10 h or more of “wear time.” The ActiGraph 7164 was worn at the waist, and steps were inferred from zero crossings of the instantaneous vertical acceleration versus time curve. On average, men took 10,578 ± 134 steps per day (mean ± SE) and women took 8882 ± 124 steps per day. After censoring steps to make them more similar to Yamax pedometer steps per day, the authors concluded that men took 7431 ± 129 steps per day and women took 5756 ± 120 steps per day. This procedure of censoring steps may help in harmonizing ActiGraph 7164 data and Yamax Digi-walker data. However, the ActiGraph 7164 step counts are quite similar, on average, to those obtained with the ankle-worn StepWatch which is often regarded as a suitable criterion for step counting [58].

The CANPLAY surveillance study examined a total of 5949 boys and 5709 girls (5–19 years of age) in Canada. They were recruited through their parents using random digit dialing and mailed a step counter and a data collection package. Girls were found to take fewer steps per day than boys (10,682 vs. 11,059, respectively), and to have less variability in daily step counts [59].

Studies on convenience samples of children in 13 countries have found that they typically have higher mean daily step counts than adults [60]. This finding is generally consistent with the 2008 US Department of Health and Human Services (DHHS) physical activity guidelines calling for at least 60 min of aerobic activity per day in children, whereas in adults the guidelines call for accumulating 150 min of moderate intensity physical activity per week, 75 min of vigorous physical activity per week, or a combination of the two (in bouts of 8–10 min or longer) [61]. In our view, step counters might be able to assess the likelihood that an individual is meeting the guidelines, but they cannot determine if the guidelines are being met. This is due to the inability of most step counters to measure frequency, intensity, and duration, as well as their inability to capture bicycling, swimming, and resistance training.

## Beyond Step Counting: Measurement of Gait Parameters

In addition to steps per day, pedometers can also provide information on cadence. Cadence is an important variable because it can be used to estimate walking speed and rate of energy expenditure. For instance, 30 min of continuous walking at 2.8 mph results in about 3000 steps being taken. Faster and slower walking speeds yields higher and lower cadences, respectively. A cadence of 100 steps/min corresponds to about 3.0 METs, and it has been proposed that this value could serve as a “cut-point” that reflects the lower bounds of MVPA (usually defined as 3.0–5.9 METs) [62].

However, cadence is not the same thing as step accumulation per minute, in free-living adults. Cadence can be thought of as a fairly instantaneous rate of stepping (measured over a few strides). Step accumulation per minute, on the other hand, refers to the total number of steps taken during a 1-min epoch [63]. If a person performs continuous walking for only half a minute, the cadence may be around 110 steps/min while they are walking, but the steps accumulated over a 1-min period will only be 55 steps/min. Thus, it is difficult to determine cadence when using 1-min epochs in free-living individuals, due to the presence of brief, intermittent bouts of walking. Another thing to consider is that while the relationship between stepping rate and energy expenditure has been quantified for walking/running, there appears to be a different relationship between these variables when other activities are performed. However, it seems reasonable to suggest that step accumulation rates can provide a crude approximation of the intensity level. For instance, the StepWatch uses the step accumulation rates to classify physical activity intensity into one of three zones (low, medium, and high activity).

Weyand et al. [64] used a shoe-mounted accelerometer to measure the foot–ground contact time, or “stance time.” They observed that foot–ground contact time is inversely related to speed of locomotion in humans, as well as in animals. They then developed a formula using foot–ground contact time to predict speed over a wide range of speeds, from slow walking to sprint running. The same group of researchers has shown that three variables (speed of locomotion, body weight, and an individual’s height) can be used to predict energy expenditure (EE) with a high degree of accuracy [65]. Taken together, this implies that extremely accurate estimates of EE are possible with a shoe-mounted device and simple anthropometric measurements.

## Potential for Integrating Step Counting into Medical Practice

Wearable medical devices are now being designed for use in clinical research settings. At least one step counter (StepWatch) has received US Food and Drug Administration (FDA) clearance as a class 2 medical device for use in research. The FDA is concerned about the safety, precision, and claimed benefits of such devices. However, unlike other medical devices that assess vital signs and clinical biomarkers (e.g., blood pressure monitors, pulse oximeters, and blood glucose monitors) at one point in time, wearable physical activity monitors assess a human behavior and must be worn continuously (or at least during all waking hours) for extended periods (e.g., 3–7 days) to provide useful information.

The ability of wearable devices to continuously store vast amounts of information on small, inexpensive computer chips has fundamentally changed the field of physical activity assessment. It alleviates concerns about physical activity questionnaires being too subjective, and people being unable to recall how much incidental activity they performed over the course of a day. Step counters have not yet become common in clinical practice, but in the future steps/day could be regarded as a vital sign that conveys important health information, and wearable medical devices could be integrated into the standard-of-care for treatment of certain diseases.

In order for step counting to become a standard component of medical care, several things would need to occur:

- Longitudinal, prospective cohort studies must demonstrate that daily stepping predicts the incidence of future disease
- Steps per day must be a rigorously validated metric, harmonized across multiple step-counting devices
- FDA clearance procedures must be established for wearable medical devices that count steps
- Health Information Privacy and Portability Act (HIPPA)-compliant file structures must be used
- Step data must be integrated seamlessly into the electronic medical record (EMR).

Reimbursement codes for objective assessment of physical activity using wearable medical devices would also speed the adoption of step counting in medical care.

## Summary

In this article, we stated that a common definition of a step is one that involves lifting the foot or prosthetic limb off the ground, moving it to a new location, and putting it back down again. The ideal location for accurately measuring steps seems to be the ankle or foot. However, waist-mounted devices are accurate enough that they can yield useful information on the relationship between steps per day and health outcomes. Currently, more studies are needed that examine the step counting accuracy of wrist-worn devices. By using step counters in physical activity interventions, we have learned that they facilitate behavior change and can be helpful in motivating inactive individuals to increase their activity levels by about 2500 steps per day (the equivalent of walking 1 mile). The development of wearable medical devices will bring exciting new advances, as physicians seek to assess their patient’s stepping behaviors, along with vital signs and clinical disease biomarkers. These new medical devices will interface with the electronic medical record and require new levels of privacy control. High levels of accuracy, especially among older and disabled patients who walk slowly and with altered gait, will be of paramount importance for wearable medical devices.

## References

[1] Stunkard A (1960). A method of studying physical activity in man. Am J Clin Nutr.

[2] Bassett DR, Strath SJ, Welk GJ (2002). Use of pedometers to assess physical activity. Physical activity assessments for health-related research.

[3] Singer E. The measured life. In: MIT Technology review. 2011. https://www.technologyreview.com/s/424390/the-measured-life/. Accessed 12 July 2011.

[4] Statista. Number of Fitbit devices sold worldwide from 2010 to 2015. 2016. http://www.statista.com/statistics/472591/fitbit-devices-sold/. Accessed 15 July 2016.

[5] Bassett DR, Mahar MT, Rowe DA, Morrow JR (2008). Walking and measurement. Med Sci Sports Exerc.

[6] Gibbs-Smith C (1978). The inventions of Leonardo da Vinci.

[7] Dumbauld E (1946). Thomas Jefferson: American tourist.

[8] Wilson DL, Stanton L (1999). Jefferson Abroad.

[9] Perrelet Watches. History 1729–2014. https://www.perrelet.com/en/brand/history. Accessed 15 July 2016.

[10] Daniels G (1975). The Art of Breguet.

[11] Hatano Y. Pedometer-assessed physical activity: Measurement and motivations. In: Presented at 48th annual meeting of the American College of Sports Medicine (May 30–June 3), 2001, Baltimore, MD; 2001.

[12] Bassett DR, Ainsworth BE, Leggett SR, Mathien CA, Main JA, Hunter DC (1996). Accuracy of five electronic pedometers for measuring distance walked. Med Sci Sports Exerc.

[13] Bassett DR, Cureton AL, Ainsworth BE (2000). Measurement of daily walking distance-questionnaire versus pedometer. Med Sci Sports Exerc.

[14] Sequeira MM, Richardson M, Wietlisbach V, Tullen B, Schutz Y (1995). Physical activity assessment using a pedometer and its comparison with a questionnaire in a large population study. Am J Epidemiol.

[15] Bravata DM, Smith-Spangler C, Sundaram V, Gienger AL, Lin N, Lewis R (2007). Using pedometers to increase physical activity and improve health: a systematic review. JAMA.

[16] Tudor-Locke C, Johnson WD, Katzmarzyk PT (2009). Accelerometer-determined steps per day in US adults. Med Sci Sports Exerc.

[17] Bergman RJ, Bassett DR, Klein DA (2008). Validity of 2 devices for measuring steps taken by older adults in assisted-living facilities. J Phys Act Health.

[18] Crouter SE, Schneider PL, Bassett DR (2005). Spring-levered versus piezo-electric pedometer accuracy in overweight and obese adults. Med Sci Sports Exerc.

[19] Feito Y, Bassett D, Tyo B, Thompson D (2011). Effects of body mass index and tilt angle on output of two wearable activity monitors. Med Sci Sports Exerc.

[20] Tyo B, Fitzhugh E, Bassett D, John D, Thompson D (2011). Effects of body mass index and step rate on pedometer error in a free-living environment. Med Sci Sports Exerc.

[21] Grant PM, Dall PM, Mitchell SL, Granat MH (2008). Activity-monitor accuracy in measuring step number and cadence in community-dwelling older adults. J Aging Phys Act.

[22] Kanoun N (2009). Validation of the ActivPAL activity monitor as a measure of walking at pre-determined slow walking speeds in a healthy population in a controlled setting. Age (Years)..

[23] Foster RC, Lanningham-Foster LM, Manohar C, McCrady SK, Nysse LJ, Kaufman KR (2005). Precision and accuracy of an ankle-worn accelerometer-based pedometer in step counting and energy expenditure. Prev Med.

[24] Karabulut M, Crouter SE, Bassett DR (2005). Comparison of two waist-mounted and two ankle-mounted electronic pedometers. Eur J Appl Physiol.

[25] Mudge S, Stott NS, Walt SE (2007). Criterion validity of the StepWatch Activity Monitor as a measure of walking activity in patients after stroke. Arch Phys Med Rehabil.

[26] Hickey A, John D, Sasaki JE, Mavilia M, Freedson P (2016). Validity of activity monitor step detection is related to movement patterns. J Phys Act Health.

[27] Hoodless DJ, Stainer K, Savic N, Batin P, Hawkins M, Cowley AJ (1994). Reduced customary activity in chronic heart failure: assessment with a new shoe-mounted pedometer. Int J Cardiol.

[28] Chen MD, Kuo CC, Pellegrini CA, Hsu MJ (2016). Accuracy of wristband activity monitors during ambulation and activities. Med Sci Sports Exerc.

[29] El-Amrawy F, Nounou MI (2015). Are currently available wearable devices for activity tracking and heart rate monitoring accurate, precise, and medically beneficial?. Healthc Inform Res.

[30] Troiano RP, McClain JJ, Brychta RJ, Chen KY (2014). Evolution of accelerometer methods for physical activity research. Br J Sports Med.

[31] Tudor-Locke C, Barreira TV, Schuna JM (2015). Comparison of step outputs for waist and wrist accelerometer attachment sites. Med Sci Sports Exerc.

[32] Tryon WW (2013). Activity measurement in psychology and medicine.

[33] Tudor-Locke CE, Bassett DR (2004). How many steps are enough? Pedometer-determined physical activity indices. Sports Med.

[34] Hatano Y (1993). Use of the pedometer for promoting daily walking exercise. Int Counc Health Phys Educ Recreat (ICHPER) J.

[35] Definition of step. Merriam-Webster Dictionary. 2016. https://www.merriam-webster.com/dictionary/step. Accessed 20 Dec 2016.

[36] Definition of step. English Oxford Living Dictionaries. 2016. https://en.oxforddictionaries.com/definition/step. Accessed 20 Dec 2016.

[37] Orendurff MS, Schoen JA, Bernatz GC, Segal AD, Klute GK (2008). How humans walk: bout duration, steps per bout, and rest duration. J Rehabil Res Dev.

[38] Crouter SE, Schneider PL, Karabulut M, Bassett DR (2003). Validity of 10 electronic pedometers for measuring steps, distance, and energy cost. Med Sci Sports Exerc.

[39] Feito Y, Bassett D, Thompson D, Tyo B (2012). Effects of body mass index on step count accuracy of physical activity monitors. J Phys Act Health.

[40] Hatano Y (1997). Prevalence and use of pedometer [article written in Japanese]. Res J Walk.

[41] Schmidt M, Cleland V, Shaw K, Dwyer T, Venn A (2009). Cardiometabolic risk in younger and older adults across an index of ambulatory activity. Am J Prev Med.

[42] Sisson S, Camhi S, Church T, Tudor-Locke C, Johnson W, Katzmarzyk P (2010). Accelerometer-determined steps/day and metabolic syndrome. Am J Prev Med.

[43] Inoue S, Ohya Y, Tudor-Locke C, Yoshiike N, Shimomitsu T (2012). Step-defined physical activity and cardiovascular risk among middle-aged Japanese: the National Health and Nutrition Survey of Japan 2006. J Phys Act Health.

[44] Katzmarzyk PT, Barreira TV, Broyles ST, Champagne CM, Chaput JP, Fogelholm M (2013). The International Study of Childhood Obesity, Lifestyle and the Environment (ISCOLE): design and methods. BMC Public Health.

[45] President’s Council on Fitness Sports and Nutrition. PALA+: activity plus nutrition. 2016. http://www.fitness.gov/participate-in-programs/pala/. Accessed 15 July 2016.

[46] Richardson CR, Newton TL, Abraham JJ, Sen A, Jimbo M, Swartz AM (2008). A meta-analysis of pedometer-based walking interventions and weight loss. Ann Fam Med.

[47] Kang M, Marshall SJ, Barreira TV, Lee JO (2009). Effect of pedometer-based physical activity interventions: a meta-analysis. Res Quart Exerc Sport.

[48] Patel MS, Asch DA, Volpp KG (2015). Wearable devices as facilitators, not drivers, of health behavior change. JAMA.

[49] Richardson CR, Mehari KS, McIntyre LG, Janney AW, Fortlage LA, Sen A (2007). A randomized trial comparing structured and lifestyle goals in an internet-mediated walking program for people with type 2 diabetes. Int J Behav Nutr Phys Act.

[50] Martinez CH, Moy ML, Nguyen HQ, Cohen M, Kadri R, Roman P (2014). Taking Healthy Steps: rationale, design and baseline characteristics of a randomized trial of a pedometer-based Internet-mediated walking program in veterans with chronic obstructive pulmonary disease. BMC Pulm Med.

[51] Krein SL, Kadri R, Hughes M, Kerr EA, Piette JD, Holleman R (2013). Pedometer-based internet-mediated intervention for adults with chronic low back pain: randomized controlled trial. J Med Internet Res.

[52] Djuric Z, Ellsworth JS, Weldon AL, Ren J, Richardson CR, Resnicow K (2011). A diet and exercise intervention during chemotherapy for breast cancer. Open Obes J.

[53] Lindbergh R (2000). Active living: on the road with the 10,000 steps program. J Am Diet Assoc.

[54] Pronk N (2003). One step at a time—the 10,000 Steps program increases physical activity. Perm J.

[55] McCormack G, Milligan R, Giles-Corti B, Clarkson JP (2003). Physical activity levels of Western Australian Adults: results from the adult physical activity survey and pedometer study.

[56] Inoue S, Ohya Y, Tudor-Locke C, Tanaka S, Yoshiike N, Shimomitsu T (2011). Time trends for step-determined physical activity among Japanese adults. Med Sci Sports Exerc.

[57] Inoue S, Takamiya T, Yoshiike N, Shimomitsu T. Physical activity among the Japanese: results of the National Health and Nutrition Survey. In: Prevention CfDCa, editor. Proceedings of the international congress on physical activity and public health; 17–20 April 2006. Atlanta, GA: U.S. Department of Health and Human Services; 2006. p. 79.

[58] Feito Y, Bassett DR, Thompson DL (2012). Evaluation of activity monitors in controlled and free-living environments. Med Sci Sports Exerc.

[59] Tudor-Locke C, Craig CL, Cameron C, Griffiths JM (2011). Canadian children’s and youth’s pedometer-determined steps/day, parent-reported TV watching time, and overweight/obesity: the CANPLAY Surveillance Study. Int J Behav Nutr Phys Act.

[60] Beets MW, Bornstein D, Beighle A, Cardinal BJ, Morgan CF (2010). Pedometer-measured physical activity patterns of youth: a 13-country review. Am J Prev Med.

[61] U.S. Department of Health and Human Services. Physical Activity Guidelines for Americans. 2008. http://www.health.gov/PAGuidelines/Report/Default.aspx. Accessed 17 Nov 2008.

[62] Tudor-Locke C, Rowe DA (2012). Using cadence to study free-living ambulatory behaviour. Sports Med.

[63] Dall PM, McCrorie PR, Granat MH, Stansfield BW (2013). Step accumulation per minute epoch is not the same as cadence for free-living adults. Med Sci Sports Exerc.

[64] Weyand P, Kelly M, Blackadar T, Darley J, Oliver S, Ohlenbusch N (2001). Ambulatory estimates of maximal aerobic power from foot-ground contact times and heart rates in running humans. J Appl Physiol.

[65] Ludlow LW, Weyand PG (2016). Energy expenditure during level human walking: seeking a simple and accurate predictive solution. J Appl Physiol (Bethesda, Md: 1985).

